# Therapeutic DNA Vaccines against HPV-Related Malignancies: Promising Leads from Clinical Trials

**DOI:** 10.3390/v14020239

**Published:** 2022-01-25

**Authors:** Jianming Tang, Mingzhu Li, Chao Zhao, Danhua Shen, Lei Liu, Xiujun Zhang, Lihui Wei

**Affiliations:** 1 Aeonvital Biomedical Research Institute, Beijing 102208, China; leiliu@aeonvital.com (L.L.); Azhang@aeonvital.com (X.Z.); 2 Department of Gynecology and Obstetrics, Peking University People’s Hospital, Beijing 100033, China; mingzhu1815@163.com (M.L.); 0062034740@bjmu.edu.cn (C.Z.); shenpath59@163.com (D.S.); weilhpku@163.com (L.W.)

**Keywords:** HPV, DNA vaccine, epitope, cervical cancer, squamous intraepithelial lesions

## Abstract

In 2014 and 2021, two nucleic-acid vaccine candidates named MAV E2 and VGX-3100 completed phase III clinical trials in Mexico and U.S., respectively, for patients with human papillomavirus (HPV)-related, high-grade squamous intraepithelial lesions (HSIL). These well-tolerated but still unlicensed vaccines encode distinct HPV antigens (E2 versus E6+E7) to elicit cell-mediated immune responses; their clinical efficacy, as measured by HSIL regression or cure, was modest when compared with placebo or surgery (conization), but both proved highly effective in clearing HPV infection, which should help further optimize strategies for enhancing vaccine immunogenicity, toward an ultimate goal of preventing malignancies in millions of patients who are living with persistent, oncogenic HPV infection but are not expected to benefit from current, prophylactic vaccines. The major roadblocks to a highly efficacious and practical product remain challenging and can be classified into five categories: (i) getting the vaccines into the right cells for efficient expression and presentation of HPV antigens (fusion proteins or epitopes); (ii) having adequate coverage of oncogenic HPV types, beyond the current focus on HPV-16 and -18; (iii) directing immune protection to various epithelial niches, especially anogenital mucosa and upper aerodigestive tract where HPV-transformed cells wreak havoc; (iv) establishing the time window and vaccination regimen, including dosage, interval and even combination therapy, for achieving maximum efficacy; and (v) validating therapeutic efficacy in patients with poor prognosis because of advanced, recurrent or non-resectable malignancies. Overall, the room for improvements is still large enough that continuing efforts for research and development will very likely extend into the next decade.

## 1. Introduction: The Need for Developing Therapeutic, Anti-HPV Vaccines

Persistent infection with oncogenic types of human papillomavirus (HPV), including HPV-16, -18, -31, -33, -45, -52, -58 and close to 20 others, predisposes patients to cervical, penile, vulvar, vaginal, anal, and oropharyngeal malignancies [1,2,3,4,5,6,7,8,9]. Globally, these HPV-associated malignancies account for 4.5% of all human cancers, with an estimated annual case burden between 500,000 and 600,000 since 2012 [2,10]. Cervical cancer is also the second most common malignancy in women and disproportionally affects developing countries [2,11,12,13]. Although two prophylactic vaccines, Gardasil (introduced in 2006) [14] and Cervarix (in use since 2009) [15], are highly effective in preventing new infections with oncogenic HPV-16 and -18, as well as low-risk HPV-6 and -11, the anti-HPV immunity induced by these commercially available vaccines is driven by antigens (i.e., recombinant capsid proteins) [14,15,16] that are rarely present in HPV-transformed cells. Accordingly, millions of patients who are living with persistent, oncogenic HPV infection or HPV-associated malignancies are not expected to benefit from these existing products [17,18]. Instead, new, therapeutic anti-HPV vaccines must target other HPV proteins, especially E6 and E7 that are the primary oncogenic factors [19,20,21,22].

**Figure 1:**
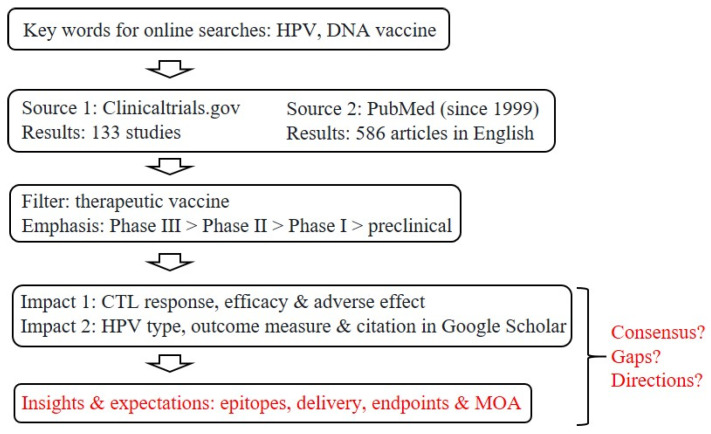
Strategies for screening and filtering current literature for a focused review. Given the large numbers of clinical trials and related literature, as already captured by two major public databases, our goal here is to summarize the current status of research and development toward therapeutic, anti-HPV DNA vaccines, with a focus on insights and expectations, as well as questions about potential consensus, remaining gaps and future directions for refinements or improvements. CTL, cytotoxic T-lymphocyte; MOA, mechanism of action.

The roads to therapeutic, anti-HPV vaccines have seen a steady flow of traffic in the past two decades, as reflected by the large number of research articles directly related to this topic: a survey conducted in November 2021 revealed 73 PubMed papers in 1999–2004, 165 in 2005–2009, 120 in 2010–2014, and 228 in 2015–2021 (Figure 1). An initial screening of clinical trials registered at ClinicalTrials.gov yielded more than 130 entries related to therapeutic DNA vaccines (the apparent front runners).

**Figure 2:**
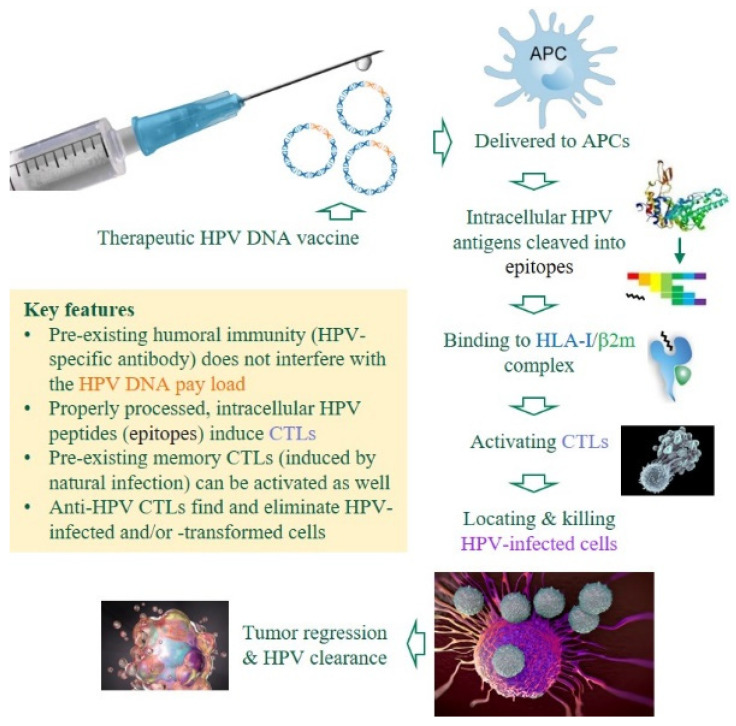
Premises and advantages of DNA-based, therapeutic anti-HPV vaccines. Unlike prophylactic HPV vaccines that induce HPV-specific antibodies to neutralize invading viruses, therapeutic DNA vaccines against HPV-induced malignancies must induce cytotoxic T-lymphocytes (CTLs) to eliminate HPV-infected or -transformed cells in patients with chronic/persistent infections [23,24]. Several critical components are color-coded. APCs, antigen-presenting cells; HLA-I, human leukocyte antigen class I (class I heavy chain); β2m, β2 microglobulin (class I light chain).

Despite all the premises and advantages of research and development (R&D) toward therapeutic, anti-HPV DNA vaccines (Figure 2), there are still no licensed products in the market, which raises three questions: (i) Is it possible to draw some consensus from past and ongoing efforts, especially clinical trials and high-impact studies? (ii) Are there gaps, roadblocks or bottlenecks that future R&D must deal with? (iii) How is the current R&D landscape likely to evolve, in terms of refinements and improvements? Our review will attempt to answer these lingering questions based on existing data in both academic and industrial arenas, with an emphasis on realistic deliverables and evidence from proof of concept research. Ramifications of the latest technological success in developing mRNA-based vaccines, which have a much shorter timeline than traditional pipelines, are also discussed to a limited extent.

## 2. Key Advantages in Developing Therapeutic, Anti-HPV Vaccines

DNA vaccines encoding tumor-specific antigens are highly attractive because of their ability to induce potent, cell-mediated immunity (as reviewed in refs. [25,26,27,28,29]). To develop therapeutic, anti-HPV vaccines, at least three major advantages are apparent. First, the HPV genome is relatively simple and small, with a circular, double-stranded DNA consisting of just nine open reading frames encoding seven early (E) proteins that govern viral replication and two late (L) proteins that form the viral capsid [6,8]. Since most (~90%) HPV infection leads to spontaneous clearance (self-cure) within two years, correlates of immune protection against these pathogens are readily defined. As such, there is a general consensus about the viral antigens and specific epitopes that should be targeted as the main immunogen in vaccine designs (Table 1). Second, a variety of clinically proven techniques, including regular Pap smears (for cervical cancer), tissue biopsies and analysis of HPV DNA, can routinely facilitate early diagnosis of HPV-associated malignancies [30,31,32]. Many patients with histologically confirmed, high-grade squamous intraepithelial lesion (HSIL, the equivalent of stage 2 and stage 3 cervical intraepithelial neoplasia, or CIN2/3), can be recruited for clinical trials without being confounded by other host factors, especially old age and compromised immune functions that a wide spectrum of aging-related cancers must confront. Indeed, most therapeutic HPV vaccines that have reached phase I clinical trials and beyond rely on HSIL regression and clearance of HPV DNA (instead of patient survival) as two key indicators of vaccine efficacy. Third, patients with persistent HPV infection and progress to malignancies are known to have immune memories against various HPV epitopes [33], some of which (e.g., E1, E2 and E5 epitopes) fall beyond E6 and E7 [7,34] but can be harnessed for enhancing vaccine efficacy, either indirectly by serving as natural immune adjuvants (through cytokine induction) or directly by triggering anamnestic immune responses when these epitopes are added to the vaccine constructs.

Additional advantages range from collaborative spirits to public interests. As HPV-associated conditions are at the intersection of infectious disease (immunology) and oncology, investigators with diverse expertise often join force to find ways to carry the field forward across regional and international boundaries [47,48,49,50]. Public interests in HPV-associated malignancies are also obvious because of their relevance to reproductive health. Indeed, a campaign launched by the World Health Organization in 2020 aimed to reduce global cervical cancer rate to less than 4 cases per 100,000 women-years through active vaccination, screening and treatment (a 90-70-90 goal) [51]. The current R&D pipeline has further benefited from the availability of various resources, including in vitro systems for the propagation of infectious viruses (e.g., the HPV-18 organotypic cultures) [52] and preclinical models for testing vaccine efficacy against two major oncogenic subtypes (HPV-16 and -18) [36,53]. The widely used TC-1, a murine tumor cell line that expresses HPV-16 E6 and E7 (a key measure of authentication), facilitates direct comparison of research data from various laboratories.

## 3. Factors That Hinder Efforts for Vaccine Development

Oncogenic HPV is well-known for its ability to evade host immune responses [54,55,56], with three HPV proteins (E5, E6 and E7) interfering with both innate (e.g., interferon) immune pathways and the antigen-processing and -presenting machinery embedded in the major histocompatibility complex (MHC). These viral attributes impair immune surveillance by cytotoxic T-lymphocytes (CTLs) and may also vary by tissue compartments, as the polycistronic HPV gene expression patterns are often site-specific [57,58] or depend on the stage of disease progression [57,59].

Poor immunogenicity of two major oncoproteins, E6 and E7, has also proved to be challenging [60]. To date, the number of known CTL epitopes defined in preclinical and clinical studies has remained small [36,37,38,39,40,41,43,44,45,46], and they tend to favor HPV-16 versus HPV-18 epitopes (Table 1). The list may change substantially if some of the predicted CTL epitopes [7,61,62,63] are validated experimentally, while epitopes beyond the E6 and E7 oncoproteins, including those in E5 [64,65,66,67,68,69,70], may help expand the spectrum of CTL targets as well [24].

The HLA class I (HLA-I) alleles known to recognize HPV CTL epitopes are also limited, with HLA-A*02 (mostly A*02:01) leading the way (Table 1), and data about other HLA-I alleles are sparse. In silico studies using immunoinformatics tools have occasionally attempted to provide pertinent information about population coverage by potential epitopes [7,61,62,63], but regional and ethnic differences in the actual distribution of HLA-I alleles and supertypes [71,72] often obscure such efforts.

## 4. Strategies for Enhancing Immunogenicity of Therapeutic DNA Vaccines

Two common tactics—codon optimization in vaccine design and electroporation in vaccine delivery—seem to be effective in enhancing the immunogenicity of HPV E6- and E7-based DNA vaccines (Table 2), and the use of nanoplasmids to evade host intracellular defense has also gained some traction [73,74]. Other modifications have been shown to: (i) prevent extracellular DNA degradation (e.g., using a nano-carrier), (ii) guide plasmid DNA to professional antigen-presenting cells (APCs), including dendritic cells that can cross-present epitopes to both CD4 and CD8 T-cells [75]; (iii) co-deliver T-helper epitopes to tune up CTL functionality [76]; (iv) improve the efficiency of DNA transfection; (v) facilitate nuclear entry of plasmid DNA, (vi) minimize the interference of plasmid or vector backbones, and (vii) combine different immunomodulatory agents to achieve synergistic effects [77,78]. These alternative strategies, however, remain at the preclinical phase of evaluation.

To overcome limited choice of T-cell epitopes in the short E6 protein (158 amino acids) and E7 (105 amino acids), there is some success in the selective introduction of point mutations that enhance CTL responses [37]. In the case of HLA-A*02-restricted epitopes, KLV9 and RTV10 (Table 1) each has a single amino acid substitution beyond the anchoring positions. The induced CTL clones are expected to readily recognize the wild-type epitopes. The use of fusion proteins should also help expand the antigenic repertoire and boost vaccine immunogenicity [24,60,86,90].

## 5. Bottlenecks in Preclinical Systems

As in other drug development, preclinical evaluations are essential to the elucidation of pharmacodynamics and pharmacokinetics, as well as toxicity/safety profiles. For the preclinical evaluation of anti-HPV vaccines, protective immunity is typically determined in mice engrafted with a murine tumor cell line, TC-1, which expresses HPV-16-specific E6 and E8 [53] or HPV-18 E6 after further modification [91]. Because MHC-TCR interaction is a prerequisite for T-cell activation, preclinical experiments must be done in C57BL/6 (B6) mice only [92]. Vaccines targeting other HPV antigens or E6 and E7 from non-HPV-16/18 types must also come up with alternative preclinical systems, including different mouse strains.

## 6. Vaccine Candidates That Have Completed Phase III Clinical Trials

**Figure 3:**
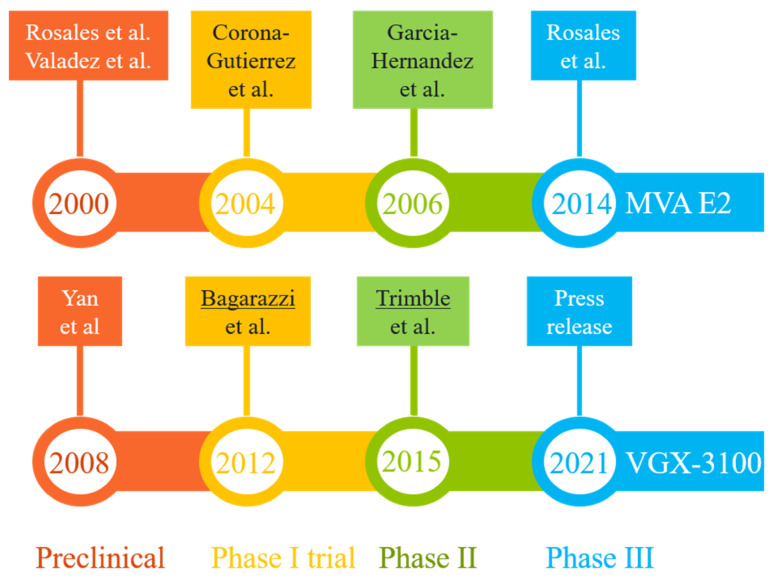
Milestones in the R&D paths of two promising vaccine candidates (MVA E2 and VGX-3100) that have completed phase III clinical trials in patients with HPV-related pre-cancerous conditions (mostly high-grade squamous intraepithelial lesions). The results from key developmental stages are readily available in peer-reviewed publications [79,80,83,93,94,95,97,98,100] or online resources [84,99]. The lead authors of two highly cited studies (more than 250 citations each) are underlined.

Two promising DNA vaccine candidates against HSIL have gone through phase III clinical trials in Mexico and U.S., respectively (Figure 3). The first, MVA E2, was intended to induce cross-protective immunity using bovine papillomavirus (BHV)-specific E2 antigen inserted into a vaccinia virus (Table 2). Performances of MVA E2 at various developmental stages (over a 14-year period) are readily available in peer-reviewed publications [79,83,93,94,95]. The largest trial so far, as reported in 2014 [83], recruited 1356 patients (including 1176 women) who received six tissue-specific injections of 1 × 10^7^ MVA E2 virus particles at weekly intervals. Overall, 1051 (89%) women showed complete elimination of intraepithelial lesions after treatment, and 81% women cleared oncogenic HPV [83]. Among 180 men who received MVA E2, all cleared intraepithelial lesions [83]. However, in the absence of a control group for side-by-side comparison, the actual efficacy (and its confidence interval) of this vaccination protocol could not be established. An earlier phase II trial did reveal that 31 out of 34 HSIL women (91%) who received MVA E2 either had total elimination of CIN2/3 or showed a 50% reduction in lesion sizes, with all vaccinated women having elevated CTLs and reduced HPV viral load. In comparison, 80% of women in the control (conization treatment, *n* = 20) group had total elimination of CIN2/3 without clearing HPV [95]. The 11% difference between the two patient groups was marginal, although the viral load and CTL data in the vaccination group may eventually translate to improved long-term benefits (e.g., a potential reduction in CIN recurrence rate), as around 15% of patients with surgical procedures may have persistent/recurrent CIN in follow-up visits [96].

The second therapeutic candidate, VGX-3100, uses a mixture of two plasmids containing codon-optimized sequences corresponding to the E6 and E7 genes of HPV-16 and -18 (Table 2). The four developmental stages (preclinical and clinical evaluations) (Figure 3) lasted 13 years [80,84,97,98,99,100]. Data from the latest phase III trial (the REVEAL 1 Study), in the form of a press release in March 2021 [84], indicated that this vaccine met its primary endpoint in a modified intent-to-treat (mITT) analysis (i.e., excluding eight patients without sufficient results)—within the mITT subset (*N* = 193), 23.7% of 131 patients in the vaccinated group responded (with HSIL regression and HPV clearance), while 11.3% of 62 patients in the placebo group did so at week 36. The rates of non-responders in both groups were quite high when compared with results for MVA E2 [83,95], but the modest vaccination efficacy (12.4% difference, 95% confidence interval = 0.4% to 22.5%) did exceed a statistical threshold (*p* = 0.02). A concurrent phase III trial involving 198 participants (REVEAL 2) is expected to reach its primary endpoint in July 2022 [101]. Of note, the same vaccine has also shown promising phase II results in treating HPV-16/18-associated anal dysplasia [102] and vulvar dysplasia [103].

In terms of mechanism of action, the BPV E2 antigen encoded by MVA E2 is intended to induce immune responses that are cross-protective against HPV—a practice similar to the one used for the highly successful smallpox vaccine. A clear advantage with MVA E2 is the potential activation of tissue-resident T-cells around the HSIL sites (through repeated local injections). Although little is known about the repertoire of conserved CTL epitopes shared between BPV and HPV E2 proteins [104], a recent analysis of HPV-specific, tumor-inflitrating lymphocytes (TILs) from patients with head and neck cancer does reveal that E2, along with E5, can be prominent targets for therapeutic vaccines as both antigens are rich with experimentally verified CTL epitopes [34]. In contrast, VGX-3100 uses naked plasmids to deliver HPV E6 and E7 antigens that differ among HPV types, coupled with a proprietary delivery system for needle-free electroporation, a technique that is known to substantially enhance T-cell immunity [105,106,107,108,109]. The resulting effector T-cells, however, must travel a long way to reach their target cells before launching a strenuous battle in an unfriendly environment.

Adverse effects associated with site-specific injection of MVA E2 vaccine ranged from headache and transient fever to abdominal and joint pains that were observed at various frequencies (6% to 69%), all of which were considered mild (grade 1, no need for immediate intervention) [83,95]. For VGX-3100 that required IM injection and electroporation, the phase III trial did not identify serious adverse events related to treatment; mild to moderate adverse events were mostly self-resolving, as seen in earlier clinical trials [84].

Overall, MVA E2 and VGX-3100 differ so starkly in their design, delivery system, administration sites, dose schedules and efficacy (Table 2) that it is just impossible to draw a consensus conclusion about the right approach. The odds of getting both MVA E2 and VGX-3100 licensed for marketing are still low: the overall success rate from phase III to regulatory approval is around 50%, with oncology products on the lower extreme (<40%) [110,111].

## 7. Promising Leads from Other Vaccine Candidates That Have Gone through Clinical Trials

Two other DNA vaccines against multiple HPV types (HPV-16 and -18) have completed phase I trials in South Korea (NCT01634503) and U.S. (NCT00788164) (Table 2). The results for the GX-188E vaccine were reported in 2014 [81], while those for pBI-11 were announced seven years later [82]. Both showed positive results in terms of tolerance, CIN regression and HPV clearance, when delivered intramuscularly either alone or in modified protocols (e.g., in combination with a second vaccine). The multifaceted NCT00788164 trial is still ongoing until summer 2022.

Four additional DNA vaccine candidates target HPV-16 only (i.e., mono-specificity) in phase I or phase I/IIa trials, and their results were published between 2004 and 2021 [86,87,88,112,113]. Again, E6 and E7 antigens are the sole immunogens, and vaccines were delivered through intradermal or intramuscular injections, with the exception of a DNA tattoo vaccine [87]. Of note, two of these vaccines each has a built-in adjuvant: IL-2 in TG4001 [112] and MIP-1α in VB10.16 [114]. A third candidate, AMV002 (formerly known as NTC-HPV16-E6/E7), involves partial codon optimization that retains portions of wild-type HPV sequences to ensure a simultaneous induction of innate immune responses [115]. The refinements provided by partial codon optimization are not trivial, as immune responses to cryptic epitopes introduced by codon optimization are known to divert T-cell responses away from the intended (authentic) epitopes [116].

Assuming that these candidates will successfully complete phase II and then advance to phase III trials, the timeline for completing these phases, as set by MVA E2 and VGX-3100 (Figure 3), ranges from six to eight years. Adding the extra time (about two years) required for collecting confirmatory data and securing regulatory approval, the best scenario is to see one of these existing candidates approved in 8–10 years. In other words, it may take another decade for these ongoing pipelines to deliver a final product.

## 8. Directions for Further Refinements

Therapeutic vaccine candidates that work well against HSIL (Table 2) may not live up to expectations for more advanced malignancies that are immunosuppressive and highly heterogeneous in nature [27,117]. In the worst scenario, several apparent bottlenecks (Table 3) may force new R&D efforts back to square one: the selection of protective CTL epitopes that are endogenous in HPV-infected and -transformed cells. For example, a recent study provides convincing evidence that multiple CTL epitopes in HPV E5 may also serve as prominent targets for therapeutic vaccines [34].

In the classic immunological toolbox, proper use of immune adjuvants improves the magnitude, breadth and durability of immune responses to protein-based vaccines [120], but the choice and formulation of adjuvants for enhancing CTL responses [121] or inducing polyfunctional CD4-cells to aid efficient and durable anti-tumor immunity [122] are not always clear. Activation of natural killer (NK) cells that prime dendritic cells toward a cross-talk with both CD4 and CD8 T-cells [123] is another worthy avenue, but reiterative assessment of these cellular mechanisms will undoubtedly further delay the R&D progress.

A new frontline in cancer immunotherapy focuses on the direct modulation of the tumor microenvironment [124,125]. In particular, T-cell inhibitory receptors can be blocked by commercially available antibodies against several T-cell checkpoint inhibitors (e.g., PD-1 and PD-L1) [126,127,128,129]. Cultivation of TILs for adoptive cell therapy is another area of active research [130,131,132] that may eventually translate to combination therapy [133].

The success of new clinical trials may further depend on biomarkers of cancer invasiveness and prognosis, as patient enrollment and data analyses can be tweaked to maximize therapeutic outcomes [134,135,136,137,138]. Indeed, clinical trials using biomarkers for patient selection seem to have a much higher success rate than trials without biomarkers [110,111].

## 9. Ramifications of Recent Success in the Development of mRNA Vaccines

The COVID-19 pandemic has brought a drastic change to the landscape of vaccine R&D [139] after two preventive mRNA vaccines against SARS-CoV-2 infection beat all the DNA vaccines in receiving a green light first for emergency use authorization and then getting a formal approval by the US Food and Drug Administration for large scale use in adults and adolescents [140,141,142]. Early perceptions and concerns about acute and chronic adverse effects have been mostly dismissed [143,144,145], even for young children who are 5–11 years old [146]. The overwhelming and paradigm-shifting successes seen with mRNA vaccines in the Western world force all R&D teams to at least reckon with the idea that existing mRNA vaccine platforms can be geared toward the rapid and cost-effective development of the next-generation therapeutic vaccines of medical and veterinary importance.

Much effort and many resources are being redirected to these new, spot-lighted avenues, but one must first and foremost recognize that access to mRNA vaccines is still limited to a handful of nations where logistics with mRNA vaccine manufacturing, transport and storage are well within their reaches. Unless mRNA vaccines and the nanotechnology (lipid nanoparticle) behind them [119,147,148] can be reformulated to ensure mRNA integrity at ambient temperatures, preferably for weeks or even months, they are simply unpractical in resource-limiting communities. Thus, stable and cost-effective DNA vaccines that require less stringent conditions for the manufacturing process and also have a much longer shelf-life than mRNA vaccines still have important roles to play in the foreseeable future.

## 10. Conclusions

Following decades of heroic efforts, a steady progress in various R&D pipelines for developing therapeutic DNA vaccines against HPV-associated malignancies still falls short of delivering a final (licensed) product to advance patient care. Two leading candidates have shown modest efficacy in late-stage clinical trials, but they share no further similarity to allow any other consensus, even about the very basics of target immunogens and delivery systems. The lessons learned from these and other candidate vaccines with clinical data, along with analyses of HPV-specific TILs, point to multiple gaps and bottlenecks that remain challenging. As such, the field may require rounds of reiterative evaluation of alternative strategies for optimal results.

## Figures and Tables

**Table 1:** Oncogenic HPV-derived epitopes that are known to induce or enhance cytotoxic T-lymphocyte (CTL) responses in preclinical and clinical studies.

Host	HPV Type	CTL Epitope	CTL Epitope Sequence	MHC Restriction	Reference(s)
Mouse	HPV-16	E6 (aa 48–57)	EVYDFAFRDL (EVL10)	H-2K	PMC479075 [35]
Mouse	HPV-16	E7 (aa 49–57)	RAHYNIVTF (RAF9)	H-2D	PMID: 7,690,326 [36]
Human	HPV-16	E6 (aa 11–19)	KLPQLCTE*V* (KL9V) ^b^	HLA-A*02	PMC5444324 [37]
Human	HPV-16	E6 (aa 29–37) ^a^	TIHDIILEC (TIC9)	HLA-B*48	PMC1182184 [38]
Human	HPV-16	E6 (aa 29–38) ^a^	TIHDIILECV (TIV10)	HLA-A*02	PMC1797519 [39]
Human	HPV-16	E6 (aa 31–38) ^a^	HDIILECV (HDV8)	HLA-B*40	PMC1797519 [39]
Human	HPV-16	E6 (aa 52–61)	FAFRDLCIVY (FAY9)	HLA-B*35, -B*57	PMID: 15,358,648 [40], PMC1182184 [38]
Human	HPV-16	E6 (aa 72–80)	KISEYRHYC (KIC9)	HLA-A*02	PMC5444324 [37]
Human	HPV-16	E6 (aa 90–99)	Q*L*YNKPLCD*V* (QLV10) ^b^	HLA-A*02	PMC5444324 [37]
Human	HPV-16	E7 (aa 11–19)	YMLDLQPET (YMT9)	HLA-A*02	PMC5444324 [37]
Human	HPV-16	E7 (aa 11–20)	YMLDLQPETT (YMT10)	HLA-A*02	PMID: 7,538,538 [41]
Human	HPV-16	E7 (aa 7–15)	TLHEYMLDL (YLL9)	HLA-A*02, -B*48	PMID: 15,358,648 [40], PMC5444324 [37]
Human	HPV-16	E7 (aa 61–69)	CDSTLRLCV (CDV9)	HLA-A*24	PMID: 21,918,960 [42]
Human	HPV-16	E7 (aa 67–76)	LCVQSTHVDI (LCI10)	HLA-A*24	PMID: 21,918,960 [42]
Human	HPV-16	E7 (aa 77–86)	RTLEDLLMG*V* (RTV10) ^b^	HLA-A*02	PMC5444324 [37]
Human	HPV-16	E7 (aa 79–87)	LEDLLMGTL (LEL9)	HLA-B*60	PMID: 15,358,648 [40]
Human	HPV-16	E7 (aa 82–90)	LLMGTLGIV (LLV9)	HLA-A*02	PMID: 7,538,538 [41]
Human	HPV-16	E7 (aa 86–93)	TLGIVCPI (TLI8)	HLA-A*02	PMID: 7,538,538 [41]
Human	HPV-18	E6 (aa 13–21)	KLPDLCTEL (KLL9)	HLA-A*02	PMID: 11,300,474 [43]
Human	HPV-18	E6 (aa 36–44)	KTVLELTEV (KTV9)	HLA-A*02	PMID: 11,300,474 [43]
Human	HPV-18	E6 (aa 50–58)	ELTEVFEFA (ELA9)	HLA-A*02	PMID: 11,300,474 [43]
Human	HPV-18	E6 (aa 54–62)	VVYRDSIPH (VVH9)	HLA-A*11	PMID: 19,738,415 [44]
Human	HPV-18/45	E6 (aa 67–75)	KCIDFYSRI (KCI9)	HLA-A*02	PMID: 16,353,149 [40]
Human	HPV-18	E6 (aa 84–92)	SVYGDTLEK (SVK9)	HLA-A*11	PMID: 19,738,415 [44]
Human	HPV-18	E7 (aa 7–15)	TLQDIVLHL (TLL9)	HLA-A*02	PMID: 11,300,474 [43]
Human	HPV-18	E7 (aa 81–95)	DDLRAFQQLFLNTLS (DDS15) ^c^	HLA-A*02, *11, *24 & 33	PMC4145224 [45]
Human	HPV-18	E7 (aa 86–94)	FQQLFLNTL (FQI9)	HLA-A*02	PMID: 12,569,558 [40]
Human	HPV-18	E7 (aa 88–97)	QLFLNTLSFV (QLV10)	HLA-A*02	PMID: 11,426,965 [46]
Human	HPV-18	E7 (aa 89–103)	LFLNTLSFVCPWCAS (LFS15)^c^	HLA-A*02, *11, *24 & 33	PMC4145224 [45]

^a^ Substantial overlapping in these epitope sequences. ^b^ Amino acid substitutions (bold and underlined) are introduced to enhance epitope affinity. ^c^ Each of these 15mer peptides contains multiple optimal epitopes. Abbreviations: aa, amino acid; HLA, human leukocyte antigen; MHC, major histocompatibility complex.

**Table 2:** Attributes of four therapeutic DNA vaccine candidates that have shown promising results against multiple HPV serotypes during or beyond phase I clinical trials.

Attributes	Four Promising DNA Vaccine Candidates ^a^ as of October 2021			
Vaccine name	MVA E2	VGX-3100	GX-188E	pBI-11
Backbone	Vaccinia virus Ankara	Two synthetic plasmids/pVAX	Plasmid/pGX27	pNGVL4a-Sig/E7(detox)
Encoded antigen	Cross-reactive E2 (bovine papilloma virus)	E6 & E7 (HPV-16 & -18)	E6 & E7 (HPV-16 & -18)	E6 & E7 (HPV-16 & -18)
Codon optimization	NA	Yes	Yes	Yes
Other modification	NA	Domain deletions	NA	Various mutations (e.g., C24G & E26G)
Vaccine adjuvant	NA	NA	NA	*Mtb* HSP70 ^d^
Companion vaccine ^b^	NA	NA	NA	TA-HPV ^e^ (IM)
Delivery	Injection, site-specific	IM, with electroporation	IM, with electroporation	IM
Dosage	6, weekly	3	3	2 pBI-11 + 1 TA-HPV
*N* in phase I trial	36 women [79]	18 women [80]	9 women [81]	30 women ^f^ [82]
Phase I registration ID	Not applicable	NCT00685412	NCT01634503	NCT00788164 ^g^
Trial site(s)	Mexico	U.S.	South Korea	U.S.
Target condition	CIN1-3	CIN2/3	CIN3	CIN3
Trial end points	CIN resolution & HPV clearance	CIN regression & HPV clearance	CIN regression & HPV clearance	CIN regression & HPV clearance
Phase III trial	Yes, in 1176 women & 180 men ^c^	Yes, in 193 subjects (mITT)	NA	NA
Latest report	PMC4270165 [83]	Website [84]	PMID: 31,727,676 [85]	PMC7845631 [75] and NCT00788164 ^g^
License	NA	NA	NA	NA

^a^ Three additional therapeutic DNA vaccines against HPV-16 alone (mono-specificity) [86,87,88] have reached phase I trial as well, as discussed in the text. ^b^ As used in various combinations. ^c^ No randomization and no placebo control. ^d^ Heat shock protein 70 helps direct vaccine immunogen to dendritic cells [75]. ^e^ TA-HPV is a recombinant vaccinia virus expressing E6 and E7 for HPV-16 and -18 [89], being tested in various treatment groups. ^f^ Split into four treatment groups. ^g^ Ongoing until July 2022. Abbreviations: CIN, cervical intraepithelial neoplasia; IM, intramuscular; mITT, protocol-defined modified intention to treat group; *Mtb*, *Mycobacterium tuberculosis*; NA, not applicable.

**Table 3:** Summary of key points drawn from past and ongoing R&D toward a DNA-based, anti-HPV vaccine.

R&D Staging	Bottleneck(s)	Directions for Improvement
Vaccine design	Limited choice of suitable CTL epitopes ^a^	Testing fusion proteins that expand the coverage of CTL epitopes
Delivery	Not always efficient, with few adjuvants	Using LNP and nanoplasmids; directing APC-specific gene expression ^d^
Preclinical system	Poor coverage (mostly HPV-16-related) ^b^	Rendering TC-1 to also express HPV-18-derived E6 and E7; developing in vitro systems for rapid assessment of immunogenicity
Clinical trials	Limited efficacy data for cancer patients ^c^	Comparing monotherapy versus combination therapy for late-stage patients with poor prognosis

^a^ Especially in the two main HPV oncoproteins, E6 and E7 (as shown in Table 1). ^b^ Can be expanded to cover HPV-18 (Table 1). ^c^ To date, most trials have involved precancer patients only (Table 2). ^d^ As for cell-specific gene editing (CRISPR-Cas9) [118]. Abbreviations: APC, antigen-presenting cell; LNP, lipid nanoparticle (~80–200 nm in diameter) [119].

## Data Availability

The original data are derived from cited literature, which are available from PubMed and websites. The corresponding author does have a private collection (full text) of cited literature that can be shared if necessary.
